# Digital Pathology Archives as Long-Term Clinical Memory: A Self-Sovereign Identity-Based Governance Model

**DOI:** 10.3390/healthcare14182892

**Published:** 2026-09-08

**Authors:** Asuman Kilitci, Arzu Kilitci Calayır

**Affiliations:** 1 Department of Pathology, Faculty of Medicine, Duzce University, 81620 Duzce, Türkiye; 2 Department of Computer Engineering, Faculty of Engineering, Istanbul Gedik University, 34876 Istanbul, Türkiye; arzu.kilitci@gedik.edu.tr

**Keywords:** digital pathology, long-term clinical memory, self-sovereign identity, patient-managed access, health data governance, verifiable credentials, dynamic consent, blockchain

## Abstract

**Background/Objectives**: Digital pathology is transforming diagnostic workflows through whole-slide imaging (WSI), digital archiving, artificial intelligence (AI)-assisted analysis, and remote consultation. The existing literature only partly addresses patient access to pathology records and cross-institutional sharing within a holistic governance framework. This study proposes a Self-Sovereign Identity (SSI)-based conceptual governance model that treats digital pathology archives as components of long-term clinical memory. Here, clinical memory refers to a pathology-specific information layer that preserves record context, provenance, integrity, and longitudinal links across time and institutions while supporting governed reuse and legally bounded patient-managed access. **Methods**: Eighty publicly available complaints from the Şikayetvar platform were examined using codebook-based thematic content analysis. Aggregated National Health Service (NHS) Written Complaints data were used to provide an illustrative external governance context. These data were not used for cross-country prevalence comparison or corroboration. **Results**: The main complaint themes were absent or inaccessible pathology results (66.3%) and records that remained unavailable despite notifications that the results were ready (55.0%). Complaint themes, the literature, regulatory sources, technical standards, and internal clinical review were synthesized while preserving different analytical functions, and 11 governance requirements were identified. **Conclusions**: Developed through Design Science Research, the model integrates verifiable credentials (VCs), a patient digital wallet, purpose- and time-limited authorization, dynamic consent and access revocation, secure off-chain storage, and blockchain-based integrity and auditing. The model was examined through an internal requirement-coverage assessment and a supplementary post hoc benchmark-oriented assessment. These analyses indicate conceptual consistency rather than independent validation. Real-world technical, clinical, usability, security, and equity validation remains necessary.

## 1. Introduction

Pathology is a critical healthcare service that supports the diagnosis, treatment, oncological follow-up, surgical decision-making, molecular testing, and second opinion processes [1,2]. In the traditional approach, pathology reports are stored in institutional information systems, whereas glass slides are kept in laboratory archives [1,3]. While this arrangement contributes to maintaining diagnostic reliability, reliance on physical archives and institution-based systems restricts patients’ access to previous pathology reports and related glass slides during subsequent care. In addition, inter-institutional referrals make continuity of care and reuse of pathology history difficult [4,5,6].

Digital pathology expands the traditional pathology workflow through whole-slide imaging (WSI), digital reporting, remote consultation, digital archiving, image management systems, and artificial intelligence (AI)-powered analysis. WSI technology enables the conversion of physical glass slides into high-resolution digital images, allowing these images to be used for education, research, quality control, telepathology, diagnostic support, and large-scale computational analysis [3,7,8]. Recent studies demonstrate that digital pathology, when combined with AI, holds significant potential in terms of diagnostic accuracy, precision oncology, and computational pathology [2,7,9]. However, the transition to digital pathology should not be limited to a mere technical shift from the microscope to the screen. This transformation is a multidimensional process that requires high-capacity digital infrastructure, secure storage, quality control, regulatory compliance, sustainable institutional workflows, and data governance [10,11,12].

The transition to digital pathology raises new management questions related to access to pathology records. These questions concern the purpose, permission, duration, and mechanisms by which access can be verified. Digitizing pathology reports and WSI files alone does not guarantee meaningful access, portability, retrievable sharing, or integrity verification. Digital pathology improves laboratory workflows and diagnostic processes. However, institution-centric, fragmented, and platform-dependent systems still limit patients’ ability to access records, manage sharing, reclaim access, and verify integrity [4,6,13].

This article addresses the governance of digital pathology archives held within institutional data structures as long-term clinical memory with patient-managed access. In this context, governance is understood as the integrated management of cross-institutional access to pathology records, authorization, consent to data sharing, access revocation, portability, verification, privacy, and auditability.

The key contribution of this study is a conceptual governance model based on self-sovereign identity (SSI). The model demonstrates how inter-institutional access to pathology reports, WSI files, and related metadata can be managed through verifiable authorization, approval, revocation, integrity, and audit mechanisms, while maintaining institutional governance of clinical records.

In this study, “clinical memory” is conceptualized as a pathology-specific, longitudinally reusable information layer that complements rather than replaces the electronic health record (EHR). Unlike conventional archival storage, which primarily preserves records, clinical memory enables the contextual relationships among pathology reports, WSI files, and metadata to be maintained over time and across institutions. It also preserves verifiable provenance, integrity, version histories, and access histories. These features enable the governed reuse of pathology information in subsequent care.

Continuity of care requires the provision of accessible and consistent clinical information across different institutions over time [14]. In digital pathology, slides or WSI files related to previous reports may be required for providing second opinions, conducting oncological follow-up, performing molecular assessments, and planning treatment [2,4,5]. Fragmented records can lead to disruptions in care and incomplete clinical decisions. Therefore, pathology records should be considered not only as institution-specific archival documents but also as reusable components of long-term clinical memory [2,8]. Patient-mediated access does not require patients to store clinical content themselves. Instead, patient-mediated access, selective sharing, and reuse in subsequent care should be supported using verifiable and auditable mechanisms in authorized repositories that store records.

Existing legal and digital health infrastructures provide a significant basis for this approach. The EHDS, GDPR, KVKK, and e-Nabız support access, legal processing, and data sharing [15,16,17,18]. However, these regulations do not fully meet the pathology-specific requirements; for example, needs such as WSI connectivity, verifiable portability, granular authorization, dynamic approval, and inter-institutional sharing are not addressed. Therefore, a complementary governance layer is needed to support patient-managed access and verifiable sharing without replacing existing clinical data repositories [6,13,19].

### 1.1. Patient-Managed Access: Definition, Scope, and Limits

In this study, patient-managed access is operationally defined by three core capabilities. Patients can (i) obtain timely access to pathology reports and record-status information and (ii) manage verifiable evidence linking a record to its authorized issuer. Where the applicable legal basis permits patient participation, they can also (iii) initiate, approve, or monitor sharing. The scope further includes the ability to (iv) define the purpose, scope, and duration of consent-based sharing and (v) withdraw consent or revoke permission for future access. Patients can also (vi) view access events and submit traceable correction or resolution requests. These functions concern access and authorization governance rather than authorship or ownership of clinical content.

In this context, the concept of “self-sovereignty” refers to the management of identity and identity-related information. It also encompasses patient participation in the authorization process. This concept does not imply absolute ownership or unilateral control over the relevant clinical records. However, the patient’s authority does not extend to modifying or deleting the content created by clinicians. Patients cannot override mandatory retention obligations or other legal grounds for data processing. Furthermore, they cannot unilaterally grant access permission in situations where doing so would conflict with patient safety, professional obligations or applicable laws. Healthcare organizations remain the authoritative custodians of the clinical records. They remain responsible for the creation, clinical validation, and correction of records through controlled changes or new versions. They are also responsible for secure storage, accessibility, retention, and legal disclosure [15,16,17]. Accordingly, the proposed SSI layer complements institutional governance through patient-mediated authorization, transparency, and auditability. Clinical custody, retention obligations, and medico-legal responsibilities are not transferred to the patient.

### 1.2. Research Gap, Questions, and Contributions

SSI, decentralized identifiers (DIDs), verifiable credentials (VCs), smart contracts, and blockchain-based audit mechanisms serve as building blocks for this complementary governance layer. In healthcare, SSI supports patient-centric identity management, secure data sharing, consent management, access control, and revocation of access [13,20,21]. In digital pathology, it allows for verifiable evidence regarding the existence, originator, integrity, status, and access conditions of a record. Therefore, SSI is considered here not as a delegation of institutional governance, but as a governance layer for patient-mediated authorization and verifiable sharing.

The proposed model adopts a hybrid architecture. Clinical content, including pathology reports, WSI files, annotations, and molecular findings, is stored in secure institutional or authorized repositories. The blockchain functions as a limited trust and audit layer only for hashes, timestamps, credential and authorization status, revocation events, and audit submissions. This separation allows large and sensitive records to be kept off-chain while also supporting integrity verification [13,20,21,22].

Studies focusing on digital pathology generally address WSI, AI-assisted analysis, digital archiving, standardization, and clinical integration [3,9,10,11]. Blockchain-based studies offer various mechanisms for access control, auditability, data integrity, consent management, and patient-centered interoperability in the context of general health data sharing. However, these studies also raise several risks related to privacy, scalability, regulatory compliance, and governance [23,24,25,26]. Healthcare research based on SSI, VCs, digital wallets, and smart contracts also offers important opportunities for patient-centered identity management, verifiable sharing, dynamic consent, access revocation, and the traceability of data use [13,19,20,27].

Blockchain research specific to the field of pathology offers important technical approaches to storing, protecting privacy, and ensuring the security of digital pathology data [28,29]. However, despite the increasing research into blockchain, artificial intelligence, and other next-generation technologies in diagnostic medicine, governance models that enable patient-managed access to digital pathology archives are limited in these studies. Specifically, current research lacks a pathology-specific governance model encompassing self-sovereign identity, dynamic consent, access revocation, selective disclosure, verifiable cross-institutional sharing, portability, interoperability, and auditability. To address this research gap, this study integrates a Design Science Research (DSR) approach with multi-source qualitative analysis. Within this scope, complaints published on the Şikayetvar platform that relate to pathology records in e-Nabız were thematically analyzed. NHS Written Complaints data were examined to provide a complementary official healthcare governance context. Academic literature, policy and regulatory documents, and technical standards and specifications were synthesized from conceptual, legal, and technical perspectives.

The thematic findings were translated into governance-related implications and examined in conjunction with other stakeholder groups. The analysis identified five governance gaps, which were formulated as R1–R11. These requirements were used as inputs in the design of the layered SSI-based model. Since the design was based on the same requirements, the scenario analysis was defined as an internal requirement-coverage assessment rather than an independent validation. In a separate post hoc assessment, externally defined privacy and cybersecurity domains were used as complementary criteria.

The study is guided by the following research questions:

**RQ1:** *Which problem areas are most prominent in publicly available pathology-related complaints posted on the Şikayetvar platform in terms of record access and visibility, data sharing, continuity of care, privacy, and patients’ control over access and sharing decisions?*

**RQ2:** *How do the selected NHS complaint categories illustrate governance domains relevant to digital pathology without corroborating the prevalence or generalizability of the Turkish findings?*

**RQ3:** *What governance gaps does the multi-source assessment reveal regarding the management of digital pathology archives as components of long-term clinical memory with patient-managed access, and into which governance requirements are these gaps translated?*

**RQ4:** *How can the identified governance requirements be reflected in a layered and hybrid SSI-based governance model?*

**RQ5:** *How do traditional, centralized digital, and proposed SSI-based workflows differ in their internal coverage of R1–R11, and how does the proposed model align with external privacy and cybersecurity benchmarks?*

This study makes three key contributions. First, by considering patient-reported complaints alongside official NHS complaint categories, it demonstrates how a formal complaints monitoring system addresses concerns regarding accessibility, communication, privacy, record accuracy, delays, and pathology.

Second, the study outlines the following traceable relationship between the analytical results and the model’s design and evaluation:

Analytical basis → governance gap → governance requirement → model component → internal assessment criterion

Third, this study proposes a layered and hybrid SSI governance model. The model combines secure off-chain clinical storage, decentralized identifiers, verifiable credentials, verifiable presentations, patient digital wallets, authorization and consent policies, and blockchain-based trust and audit mechanisms. In this approach, digital pathology archives function as components of long-term clinical memory that patients can access and share through verifiable and legally restricted procedures.

The remainder of the paper is structured as follows. Section 2 reviews digital pathology archives, health data governance, SSI, and blockchain technology. Section 3 describes the research design, source-specific analytical functions, coding procedure, and internal requirement-coverage assessment. Section 4 reports the thematic findings, the illustrative NHS governance context, and five governance gaps translated into R1–R11. Section 5 presents the model, implementation challenges, and equity considerations. Section 6 reports the internal requirement-coverage and supplementary benchmark-oriented assessments. Section 7 translates the findings into policy options, implementation pathways, and validation priorities.

## 2. Literature Review

This review examines digital pathology, archive governance, blockchain-based health data governance, and SSI as the conceptual and technical foundations of the proposed model.

### 2.1. Digital Pathology

The transition to digital pathology should not be limited to the conversion of glass slides into digital images and their examination on screens. Digital pathology also requires a high-capacity infrastructure for the secure, long-term archiving and verification of WSI files. It also requires technical standards, regulatory compliance, and organizational change management [10,11,12]. Therefore, digital pathology should be understood as a sociotechnical transformation in the storage, access, sharing, verification, reuse, and governance of pathology records.

Artificial intelligence enhances the diagnostic and analytical value of digital pathology records. However, associating these records with prognosis, biomarker analysis, treatment response, and sensitive oncology creates additional requirements in terms of privacy and data management [2,7,9].

Federated learning has been used as a method for analyzing histopathology and gigapixel WSI files across institutions without centrally aggregating raw images. However, the method also carries risks related to model update leakage and poisoning [30].

### 2.2. Governance of Digital Pathology Archives

Digital pathology archives consist of WSI files, pathology reports, annotations, metadata, molecular test results, and artificial intelligence outputs. These archives require secure access, data integrity, interoperability, and long-term governance. The Digital Imaging and Communications in Medicine (DICOM) standard and its web-based service, DICOMweb, standardize the representation and transmission of medical images and their associated metadata. Health Level Seven (HL7) Fast Healthcare Interoperability Resources (FHIR) enables the sharing of structured clinical data linked to pathology reports among health information systems [31,32,33,34].

Governance requirements differ among the record classes. WSI files and imaging metadata must ensure integrity, retention, and interoperable access [31,32]. Pathology reports and their appendices require authorship, version traceability, and professional accountability. Comments, derived metrics, and AI-generated outputs must meet the requirements for citation and validation [2,7,9]. Sequential reinterpretations should be traceable and linked to the original record rather than being directly overwritten [5].

The establishment of a digital pathology archive does not guarantee patient access, data portability, or the verifiable sharing of data. Even if records are stored within an institution’s own system, patients may not be able to access them. Furthermore, patients may have difficulty transferring records to another institution or may be unable to verify the source and current status of these records [35]. Decentralized storage across institutions can also hinder the creation of a comprehensive clinical record and disrupt the continuity of care [4,14]. To support reliable reuse, the source of the report, specimen type, dates, WSI link, and version history must be preserved [5].

Pathology reports do not consist solely of laboratory findings that confirm a previously performed diagnostic procedure. They serve as sources of clinical information that can be reused in treatment, follow-up, molecular evaluation, and second-opinion processes [1,2]. For example, in the context of head and neck cancer surgery, information gaps between stages of care may adversely affect clinical coordination [36]. Within the limits of its specific clinical context, this finding supports the importance of transferring pathology data to subsequent stages of care in a traceable manner and preserving the clinical context.

The EHDS aims to enable individuals to access certain electronic health data free of charge and without delay. Additionally, patient safety or ethical considerations may cause delays [15]. The rights to access, rectify, and erase data under the GDPR are also being implemented in accordance with applicable legal requirements and health record retention obligations [16]. In Türkiye, health data are protected as special-category personal data under the Personal Data Protection Law [17]. E-Nabız provides individuals with the ability to access certain health data and manage sharing permissions [18]. However, these infrastructures are not sufficient on their own to meet pathology-specific requirements such as WSI connectivity, verifiable report portability, dynamic consent, and verifiable cross-institutional sharing.

Consistent with Section 1.1, patient-managed access operates within institutional duties concerning record integrity, retention, patient safety, and lawful processing [6,13,37].

### 2.3. Blockchain-Based Health Data Governance

Blockchain-based governance is particularly important when multiple organizations require shared, verifiable evidence regarding record integrity, authorization status, timestamps, and access events. The function of this approach differs from the digital archive layer discussed in Section 2.2: the blockchain does not replace organizational data repositories or clinical management systems. Rather, it provides a limited level of trust and oversight. However, its use brings with it privacy, scalability, interoperability, technical maturity, and regulatory risks [25,38,39,40].

Storing pathology reports or WSI files directly on the blockchain makes it difficult to ensure compliance with data minimization and deletion requirements [16,25]. Therefore, a hybrid architecture is preferred for this purpose. Clinical content is stored in secure off-chain repositories, whereas only cryptographic hashes, timestamps, and minimum status or validation references are preserved on the chain [26,41].

Similarly, integrity-focused architectures also clearly separate off-chain data processing from on-chain integrity proofs [42].

In a pathology-specific study, a proof-of-concept application was developed for the storage and sharing of digital pathology data using smart contracts and decentralized file storage [28]. In another study, a technical approach to privacy-preserving pathology data sharing among remote parties was proposed [29]. These studies contribute technical mechanisms for storage, data transfer, access security, and privacy.

Moztarzadeh et al. [43] address the requirements for a platform for digital twins using an imaging-based blockchain. Relevant requirements include autonomous decentralized identity, interoperability, governance, and security. However, decentralized identifier (DID) and verifiable credential (VC) workflows alone do not provide a sufficient process for patient-mediated consent, access revocation, and archive governance.

Current digital pathology frameworks address different self-sovereign identity (SSI) and governance components. However, these frameworks do not encompass elements such as verifiable credentials, patient digital wallets, dynamic consent, revocation of access, record version lifecycle, auditable emergency access, and requirement–model traceability. Therefore, this study integrated these components into a pathology-specific conceptual governance model.

### 2.4. Self-Sovereign Identity

SSI allows individuals to store and share verifiable evidence in a digital environment without referring to a single central identity provider for each exchange. In centralized systems, an organization manages accounts and access within its portal. Under SSI, trusted institutions continue to issue credentials and are responsible for them. The receiving organization verifies the validity and status of the issuer [13,20,44]. Therefore, SSI transforms the way identity and authorization evidence are transmitted and verified. It does not transfer clinical authorship, retention obligations, or legal responsibilities from healthcare organizations to patients.

Self-sovereign identity offers a patient-centered approach to digital identity and credential sharing. A decentralized identifier (DID) is associated with verification methods and service endpoints, but it does not by itself establish a clinical identity, verify a person, or grant data access. A verifiable credential (VC) is digitally signed evidence issued by a trusted authority. A verifiable presentation (VP) conveys selected credential claims to a verifier [13,20,44,45]. These mechanisms reduce reliance on a single identity provider without eliminating the responsibilities of issuers, verifiers, or healthcare institutions.

In this model, identity comprises the attributes and identifiers that represent an actor. Authentication establishes control over an identifier, account, cryptographic key, or credential. Authorization is a separate decision about whether an authenticated actor may access specified data for a defined purpose, scope, duration, and legal basis. Consent may inform that decision, but it is neither identity proof nor automatic authorization and is not the applicable legal basis in every context [16,17,44,45]. Governance encompasses the policies, roles, accountability, and oversight applied to these functions.

Dynamic consent mechanisms can allow patients to update their data-sharing preferences and, in cases where consent serves as a valid basis, to withdraw their consent for future uses [46]. Consent alone does not verify a requester’s identity or automatically authorize access. In the context of digital pathology, SSI can represent pathology-related rights and evidence without disclosing the full clinical record. A patient can present the credential claims requested by a verifier through a VP [44]. A pathology laboratory or hospital can issue a VC containing or referencing statements regarding the existence, issuer, timestamp, hash value, and status of a pathology report. The patient may transmit this VC or a VP derived from it to another healthcare provider. The receiving organization first verifies the presentation, issuer, and credential status. It then performs a separate authorization assessment. If access is granted, the institution may retrieve the relevant off-chain record within the approved scope. This distinction supports portability, integrity verification, selective disclosure, and patient-mediated sharing without treating identity verification, consent, and authorization as interchangeable components [13,20,21,44].

Smart contracts or other technical policy services can codify principles governing access to and sharing of pathology records. These principles can define the purposes, scope of data, duration of access, conditions for revocation, and audit requirements. These mechanisms support policy enforcement and event recording. However, they do not determine ownership of clinical records, provide a valid legal basis, or alter institutional authorization and management arrangements [16,17,27,40].

A governance model must distinguish between routine care, referrals, second opinions, research, emergencies, authorized representatives, and other third-party access. Each context must include clear rules regarding identity verification, authorization, duration, revocation, and accountability. For example, a patient may grant a second healthcare facility access to specific pathology records for a defined purpose and for a limited period, in accordance with applicable laws and institutional policies.

Self-sovereign identity is an emerging approach in healthcare. Studies highlight challenges related to scalability, usability, regulatory compliance, institutional integration, identity recovery, digital literacy, accessibility, and user acceptance [20,21,47]. Therefore, rather than positioning self-sovereign identity as a complete technological solution, this study considers SSI a governance layer that must be integrated with institutional responsibility, legal compliance, clinical workflow requirements, and ethical boundaries.

## 3. Methodology

This design-oriented study developed an SSI-based conceptual governance approach for digital pathology archives as components of patient-managed access to long-term clinical memory. The analysis encompassed five resource groups: academic literature, policy and regulatory documents, technical standards and specifications, publicly available patient complaints, and aggregated official complaint statistics.

### 3.1. Research Design

The research design consists of three interconnected phases. In the first phase, a codebook-based thematic content analysis was conducted to identify issues arising from patient reports in the Şikayetvar text corpus. NHS Written Complaints data served only as an illustrative external governance context. Academic, policy, regulatory, and technical sources contributed to analyses at the conceptual, legal, and technical levels. In the second phase, the sources were synthesized according to their specific analytical functions, and governance gaps were identified and formulated as R1–R11. In the third phase, these requirements were used as design inputs for a layered, SSI-based governance model developed through Design Science Research (DSR). During the DSR process, actors and data objects were identified to ensure traceability between requirements and the model. The process was completed with an internal requirement-coverage assessment, a clinical expert review, and model refinement.

**Figure 1:**
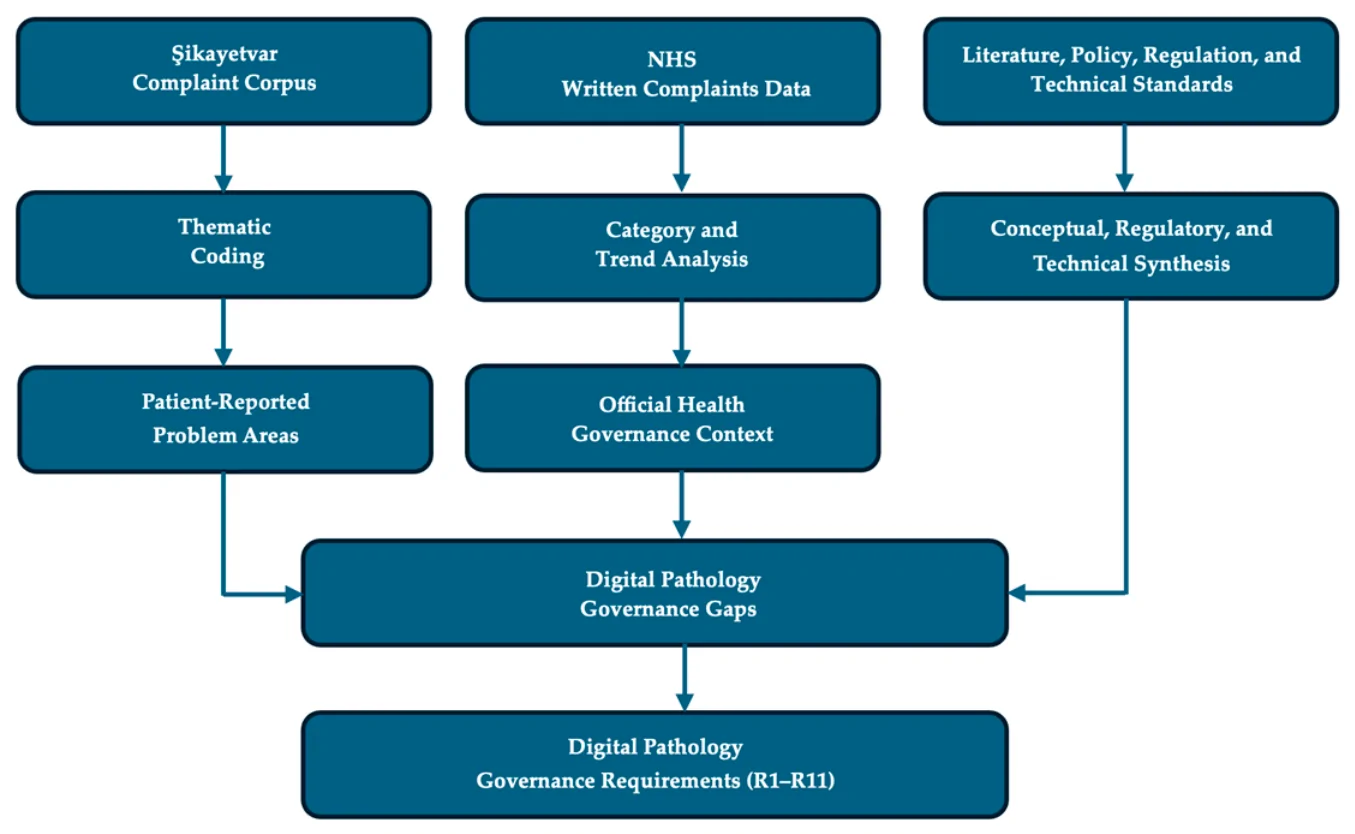
Identification of Digital Pathology Governance Gaps and Requirements.

Figure 1 outlines how resource-specific analyses are translated into governance gaps and requirements. In the study, since theme frequencies were determined descriptively through structured coding, the complaint compilation was examined with thematic content analysis based on the codebook, and the analysis results were reported [48,49].

### 3.2. Data and Document Sources

The study was based on complaints published on Şikayetvar, aggregated statistics on written complaints to the NHS, academic literature, policy and regulatory documents, and technical standards. Each source was used for a different analytical purpose. For this reason, the sources were not evaluated as statistically comparable datasets.

Public complaints posted in Şikayetvar’s “E-Nabız Pathology Results” category were analyzed to identify the issues reported by complainants regarding access, record visibility, consistency of notifications, sharing, and continuity of care [50]. The NHS complaint categories identified for the periods 2022–2023 through 2024–2025 were selected because their official classifications address record accessibility, communication, record accuracy or loss, privacy, delays in diagnosis or referral, and pathology services [51,52,53].

The academic literature provides a conceptual and technical foundation for digital pathology, health data governance, blockchain, SSI, VCs, and secure data sharing. The regulatory analysis covers the GDPR, KVKK, and EHDS. Technical standards are examined in light of their specific functions. DICOM/DICOMweb is used for the representation and sharing of medical images [31]. HL7 FHIR facilitates the sharing of structured clinical data [32]. The World Wide Web Consortium (W3C) DID and VC specifications support decentralized identity and verifiable record sharing [44,45]. Table 1 summarizes the function assigned to each source group.

### 3.3. Complaint Corpus

In June 2026, an initial screening was conducted of 83 publicly accessible complaints in the “E-Nabız Patoloji Sonucu” category on Şikayetvar [50]. Three records were excluded because they had been withdrawn, were duplicates, or contained insufficient information for thematic analysis. The final analytical corpus therefore comprised 80 complaints. Each complaint was assigned an anonymous study code for use throughout the analysis. The inclusion and exclusion criteria are presented in Table 2.

The thematic content analysis based on the codebook was conducted in three stages. First, complaint titles and texts were examined inductively in terms of recurring issues related to access, record visibility, delay, sharing, integrity, confidentiality, continuity of care, and resolution requests. These findings formed the basis for the development of the initial codebook defining T1–T11. Second, the codebook was systematically applied to all 80 complaints. Since the codes are not mutually exclusive, a single complaint may receive more than one code, and the resulting totals may exceed 80% or 100%. Third, the coded findings were synthesized under seven governance domains: access, visibility, integrity, sharing, confidentiality, continuity, and auditability. This synthesis formed the basis for governance requirements. Table 3 presents the T1–T11 codebook and operational definitions.

The coding was limited to topics explicitly stated in the complaint texts. When a statement matched more than one operational definition, all applicable codes were assigned to it. Anonymized case codes, T1–T11 assignments, and a brief coding rationale were recorded in a manually created Microsoft Excel matrix. Frequency data reflect only the distribution of themes within the self-selected complaint dataset. Initial coding for all 80 complaints was performed by one researcher. To increase analytic consistency, a second investigator re-evaluated ambiguous and multi-coded cases. The review involved repeated comparison of full complaint narratives with T1–T11 operational definitions.

Differences in interpretations were discussed until consensus was reached. The coding was not conducted as an independent double-coding study, and intercoder reliability statistics were not calculated.

### 3.4. Determination of Governance Requirements

Governance requirements were formulated using a functionally differentiated synthesis approach that preserved the analytical function of each resource. Complaint themes encompassed issues reported by patients, whereas selected NHS categories reflected relevant areas in a formal complaint tracking system. Literature and regulatory documents define conceptual and normative boundaries, whereas technical standards support interoperability and verifiability. The resulting R1–R11 requirements were used as the design inputs for the SSI-based model.

#### 3.4.1. Development of the DSR-Based SSI Governance Model

DSR provides a problem-solving approach for the development and evaluation of design outputs [54,55]. In this study, this approach was used to identify the problem context, objectives, actors, data objects, layered architecture, and fundamental governance mechanisms. Subsequently, requirement–model traceability was established. Because R1–R11 informed the model, the scenario study was framed as an internal requirement-coverage assessment of conceptual consistency. Its findings guided model refinement.

The architectural layers of the proposed model were developed based on the R1–R11 requirements. The core design principles included data minimization, interoperability, verifiability, access limited by purpose and scope, auditability, and the secure storage of clinical data.

**Figure 2:**
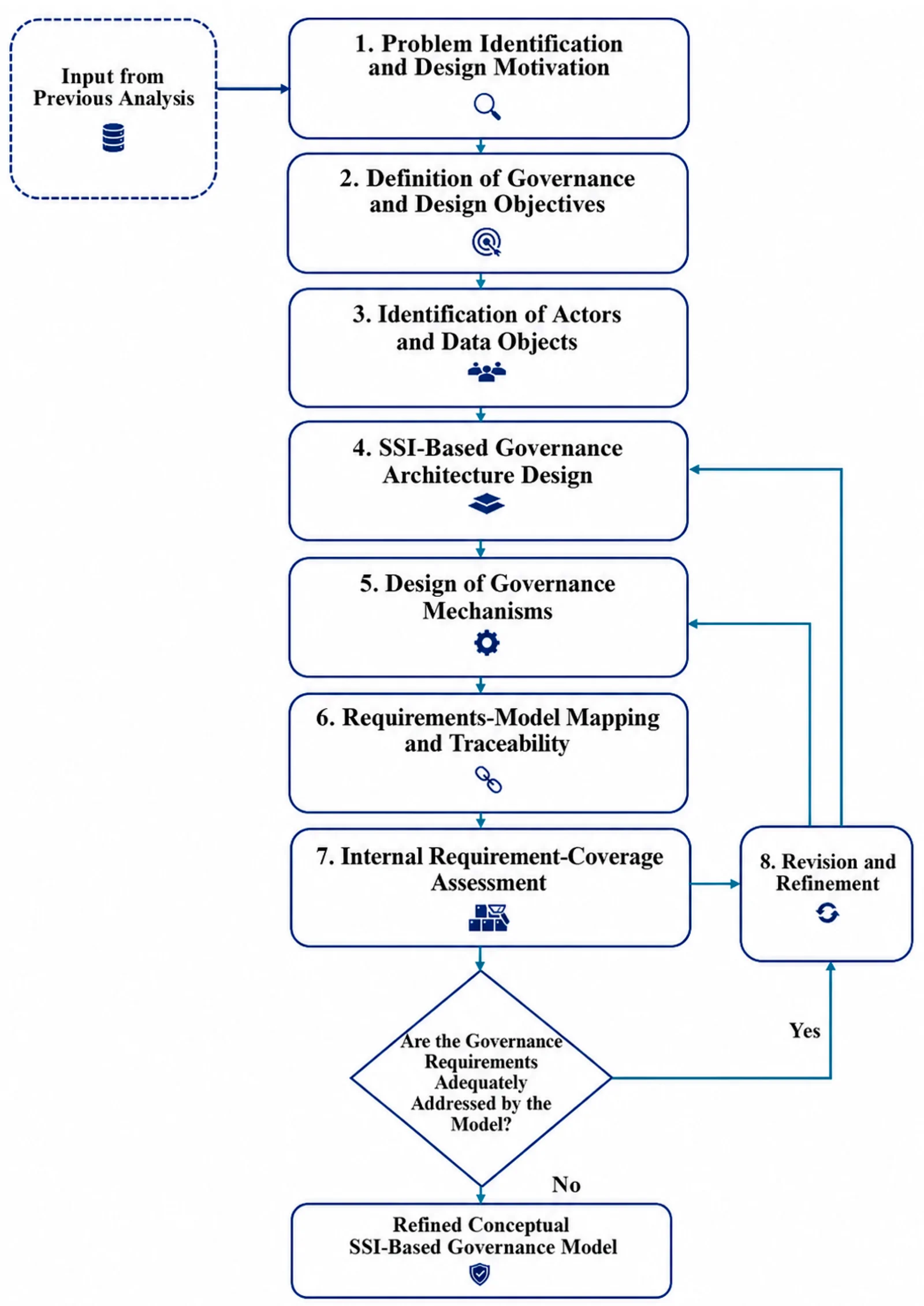
DSR-Based Development, Internal Requirement-Coverage Assessment, and Refinement of the SSI-Based Digital Pathology Governance Model.

A pathologist reviewed the model for clinical terminology and fit with digital pathology workflows. The review informed refinements to stakeholder responsibilities, terminology, and workflows. Since the pathologist is a member of the author team, this study does not constitute an external clinical validation. Figure 2 summarizes the internal requirement-coverage assessment and model refinement process. The structured internal clinical review and the subsequent model refinements are reported in Appendix A.

#### 3.4.2. Internal Requirement-Coverage and Conceptual Consistency Assessment

The proposed model was subjected to a structured internal requirement-coverage assessment based on R1–R11. This assessment considered three scenarios: the traditional pathology workflow, the centralized digital pathology workflow, and the proposed SSI-based workflow for long-term clinical memory with patient-managed access. The assessment examined whether each scenario clearly specified mechanisms corresponding to each requirement. This assessment does not constitute independent validation.

The traditional pathology workflow denotes a structure in which records are created, stored, and managed within individual institutions, whereas sharing depends primarily on manual procedures or cross-institutional exchanges upon request. The centralized digital pathology workflow describes access through centralized infrastructures, including hospital information systems, patient portals, and national health platforms. In that scenario, identity and access decisions remain governed by institutional or platform-level control.

The SSI-based workflow combines VCs, a patient digital wallet, purpose- and time-limited authorization, secure off-chain access to clinical data, and blockchain-based audit mechanisms within a single conceptual framework. Access decisions are assessed based on professional role, purpose, scope, duration, consent, or other legitimate legal grounds. Credential verification by itself does not grant automatic access to clinical content.

R1–R11 served both as design inputs and as criteria for the internal requirement-coverage assessment. Accordingly, the analysis examined requirement coverage and conceptual consistency rather than predictive, clinical, or technical validity. Each workflow was classified as ‘not addressed,’ ‘partially addressed,’ or ‘conceptually addressed.’ To reduce reliance on internally derived criteria, a separate post hoc benchmark-oriented assessment applied selected domains from the NIST Privacy Framework and the NIST Cybersecurity Framework 2.0 [56,57]. This supplementary assessment remained conceptual and did not constitute independent empirical validation. Appendix B provides the detailed rationale underlying the R1–R11 coverage classifications.

#### 3.4.3. Analytical Consistency and Traceability

Analytical consistency was supported by predefined suitability criteria, operational definitions, a structured coding matrix, investigation of ambiguous phenomena, and traceability extending from source-level findings to the requirements and model components. Evidential sources retained their separate analytical functions and were not treated as statistically comparable datasets.

#### 3.4.4. Ethical Considerations and Methodological Limitations

This study did not involve participant recruitment, interaction with participants, or intervention. No non-public clinical records or personal health data were used. Complaints from Şikayetvar were manually reviewed on publicly available pages that did not require registration. NHS data were obtained from aggregated official statistics. Usernames, direct quotations, and individual complaint links were not reported. Findings are presented only in aggregate form. The study was exempted from formal ethics approval. Because it utilizes secondary, fully anonymized data without direct human intervention or personal health identifiers. Consequently, informed consent was not applicable.

Since the Şikayetvar corpus is a self-selected sample, the theme frequencies are not indicative of clinical or societal prevalence. NHS data were used solely as an illustrative external governance context. These data were not evaluated for the purpose of making comparisons between countries or confirming the prevalence of the problems identified in Türkiye.

## 4. Findings

The themes identified from the Şikayetvar corpus highlight key problem areas in the patient experience regarding access to digital pathology records, record visibility, continuity of care, data sharing, and record reliability.

### 4.1. Thematic Findings from the Şikayetvar Corpus

**Figure 3:**
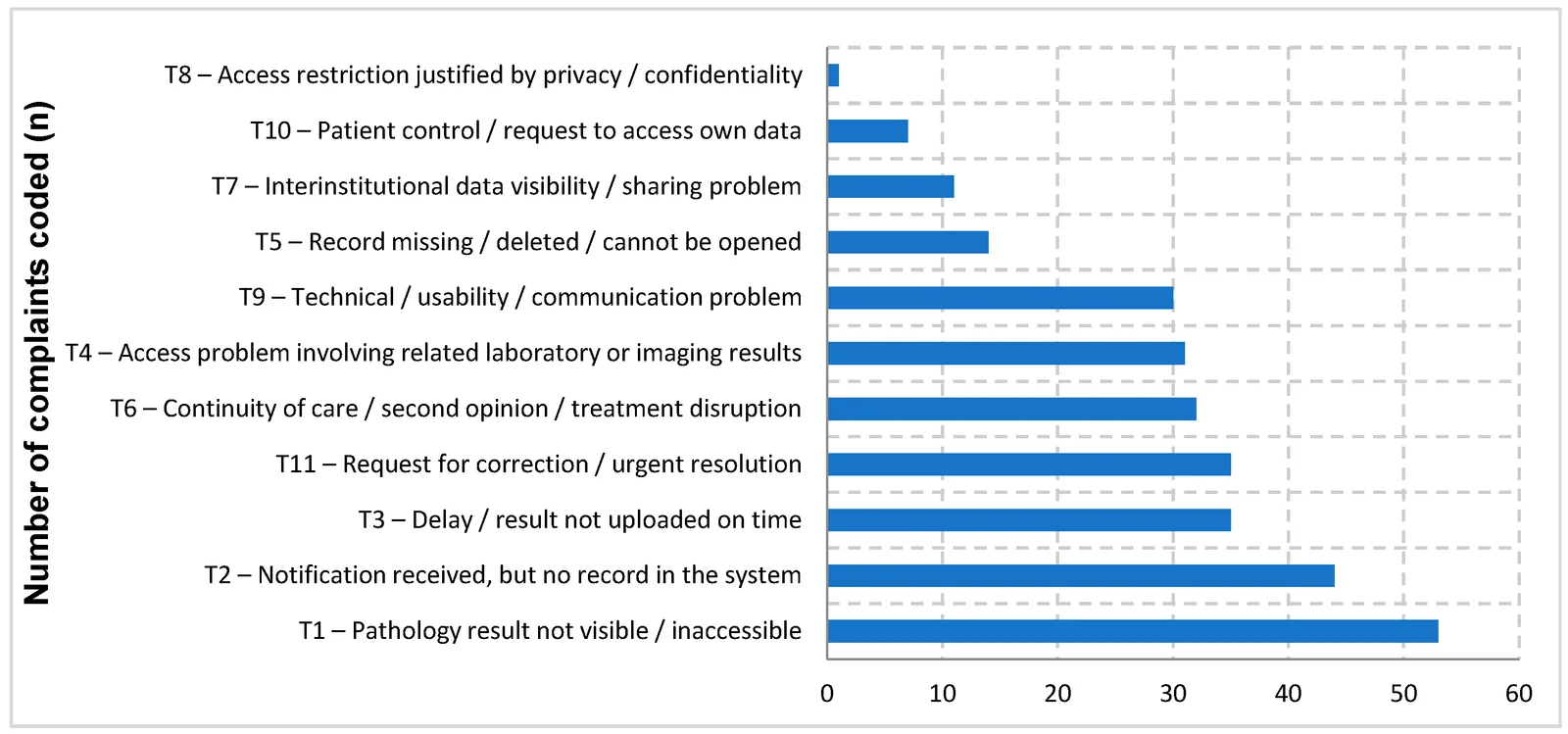
Distribution of Non-Mutually Exclusive Thematic Codes in the Şikayetvar Complaint Corpus (*n* = 80).

Figure 3 presents the frequencies of the thematic codes identified in the complaint corpus, with each complaint eligible for assignment to more than one code.

The most frequent theme was the unavailability or inaccessibility of pathology results (T1; *n* = 53; 66.3%), followed by records remaining inaccessible despite notification (T2; *n* = 44; 55.0%). Other common themes included delayed receipt of results (T3; *n* = 35; 43.8%), requests for correction or resolution (T11; *n* = 35; 43.8%), and disruptions in the continuity or second opinion processes (T6; *n* = 32; 40.0%). Difficulty accessing relevant clinical results (T4; *n* = 31; 38.8%) and technical, usability, or communication-related problems (T9; *n* = 30; 37.5%) were also frequently encountered.

Less frequently reported themes included missing, incomplete, or inaccessible records (T5; *n* = 14; 17.5%) and issues related to inter-institutional data sharing (T7; *n* = 11; 13.8%). Complaints regarding patients’ requests for access and control over their own data (T10; *n* = 7; 8.8%) and privacy-based access restrictions (T8; *n* = 1; 1.3%) were the least frequently reported issues. Overall, complaints focused on the accessibility, visibility, and timely access to records.

### 4.2. Official Governance Context Provided by NHS Written Complaints Data

**Figure 4:**
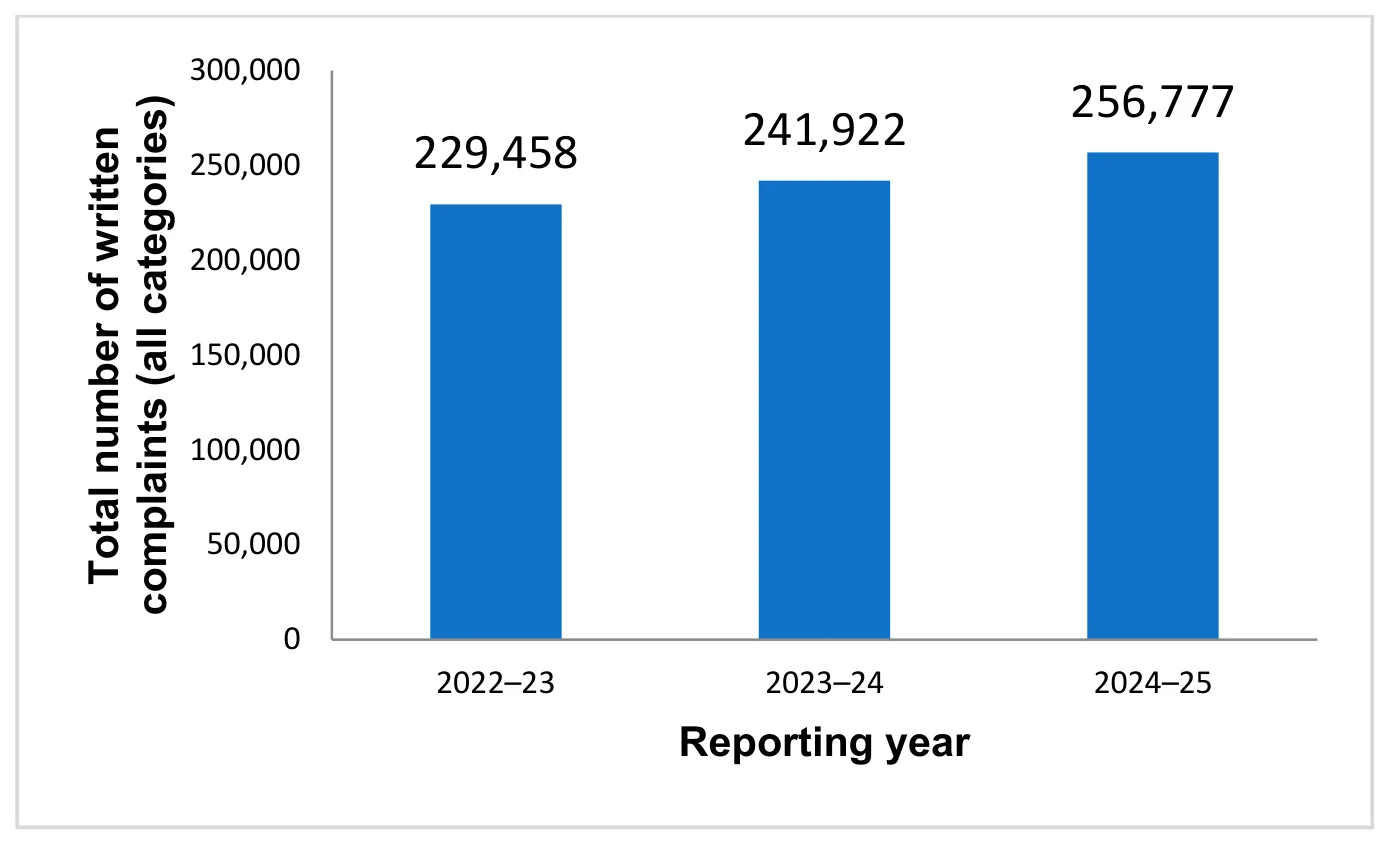
NHS Written Complaints across All Categories.

NHS written complaints data were considered only as an illustrative external governance context. They were not used for comparison or verification of prevalence levels across countries. Figure 4 shows the annual NHS totals for the three selected reporting periods.

The total number of written complaints was recorded as 229,458 for the 2022–2023 period, 241,922 for the 2023–2024 period, and 256,777 for the 2024–2025 period [51,52,53]. These figures were not interpreted as direct evidence of changes in service quality or the prevalence of problems in Türkiye.

The selected NHS categories were predetermined because they conceptually overlapped with the governance areas identified in the Şikayetvar corpus. These areas included communication, record accuracy and loss, record access, privacy, delays in diagnosis or referral, and pathology services. Table 4 presents the categories as explanatory external classification frameworks.

Among the selected NHS categories, communication had the highest number of complaints over the three periods. The number of complaints varied in categories related to record accuracy or loss, denial of access, and pathology services. These descriptive patterns illustrate how a formal complaints system can monitor governance areas relating to access, communication, integrity, and continuity.

### 4.3. Digital Pathology Governance Gaps

A functionally differentiated synthesis of complaint themes, integrated with exemplary NHS governance contexts, academic literature, policy and regulatory documents, and technical standards, revealed five key governance gaps.

**Access and data control:** Patients have limited ability to access their pathology records in a timely manner, monitor record status, and manage decisions concerning the sharing of these records.

**Record visibility and integrity:** The status, source, currency, and version history of records are often insufficiently visible and may not be verifiable.

**Cross-institutional portability and continuity of care:** The ability to share pathology reports, WSI files, and associated clinical data across institutions in an integrated and verifiable manner depends on existing integration capacity.

**Privacy, data minimization, purpose limitation, and consent management:** Mechanisms for limiting access to clinical data by purpose, scope, and duration, as well as for managing consent dynamically and revocably, may remain inadequate.

**Auditability, correction, and emergency access:** Processes for tracking access and correction requests, managing record versions, and auditing exceptional access are not sufficiently clear.

Five deficiencies corresponded to the requirements as follows: access and data control informed R1, R2, R7, and R9. Record visibility and integrity were informed by R2, R3, and R8. Portability and continuity informed R4 and R5, respectively. Confidentiality, data minimization, purpose limitation, and consent informed R6, R7, and R10. Auditability, redress, and urgent access were mentioned by R8, R9, and R11. Table 5 outlines the contribution of each resource group and separates the requirements arising from complaints from those primarily of a technical, regulatory, ethical, or clinical nature.

Together, these gaps provide the design basis for the SSI-based governance model presented in Section 5.

## 5. Proposed SSI-Based Governance Model for Digital Pathology

In this section, an SSI-based digital pathology governance model is presented, using the R1–R11 governance requirements identified during the analysis phase as design inputs. The model was developed using a DSR approach. It covers requirement–model traceability, the definition of actors and roles, core data objects, a layered governance architecture, and authorization and consent mechanisms. It also addresses access, verification, and audit processes collectively. The model’s objective is to support patient access, verifiable cross-institutional sharing, data minimization, and accountability.

### 5.1. Requirement–Model Traceability

The governance requirements R1–R11 defined in Table 5 are mapped to the relevant layers, components, and governance mechanisms of the proposed model. This mapping demonstrates how the governance issues identified through the multi-source synthesis have been incorporated into the model’s design and which mechanisms within the model address each requirement. The requirement–model relationship is presented in Table 6.

### 5.2. Actors, Roles, and Core Data Objects

An SSI-based digital pathology ecosystem adapts the roles of issuer, holder, and verifier to the digital pathology domain while accounting for clinical and institutional obligations in healthcare.

Pathologists and other authorized clinical professionals prepare, review, and sign reports and publish controlled revisions or additions. Pathology laboratories and healthcare organizations oversee record integrity, versioning, retention, and accountability and may publish pathology VCs. Archive or infrastructure providers conduct storage, retrieval, interoperability, and record-keeping activities under institutional authority; however, they do not possess independent rights to clinical content [31,32,44].

Patients or their legally authorized representatives store and present VCs/VPs, view access events, and manage applicable approval or authorization preferences. Possession of an identity document does not grant the authority to modify institutional clinical records or to assume institutional responsibility. Identity and authorizations on the identity document support verification, trust records, keys, and identity document status; however, they do not provide clinical access. Receiving organizations verify presentations and make separate authorization decisions based on the role, purpose, scope, duration, and legal basis [15,44,45].

**Figure 5:**
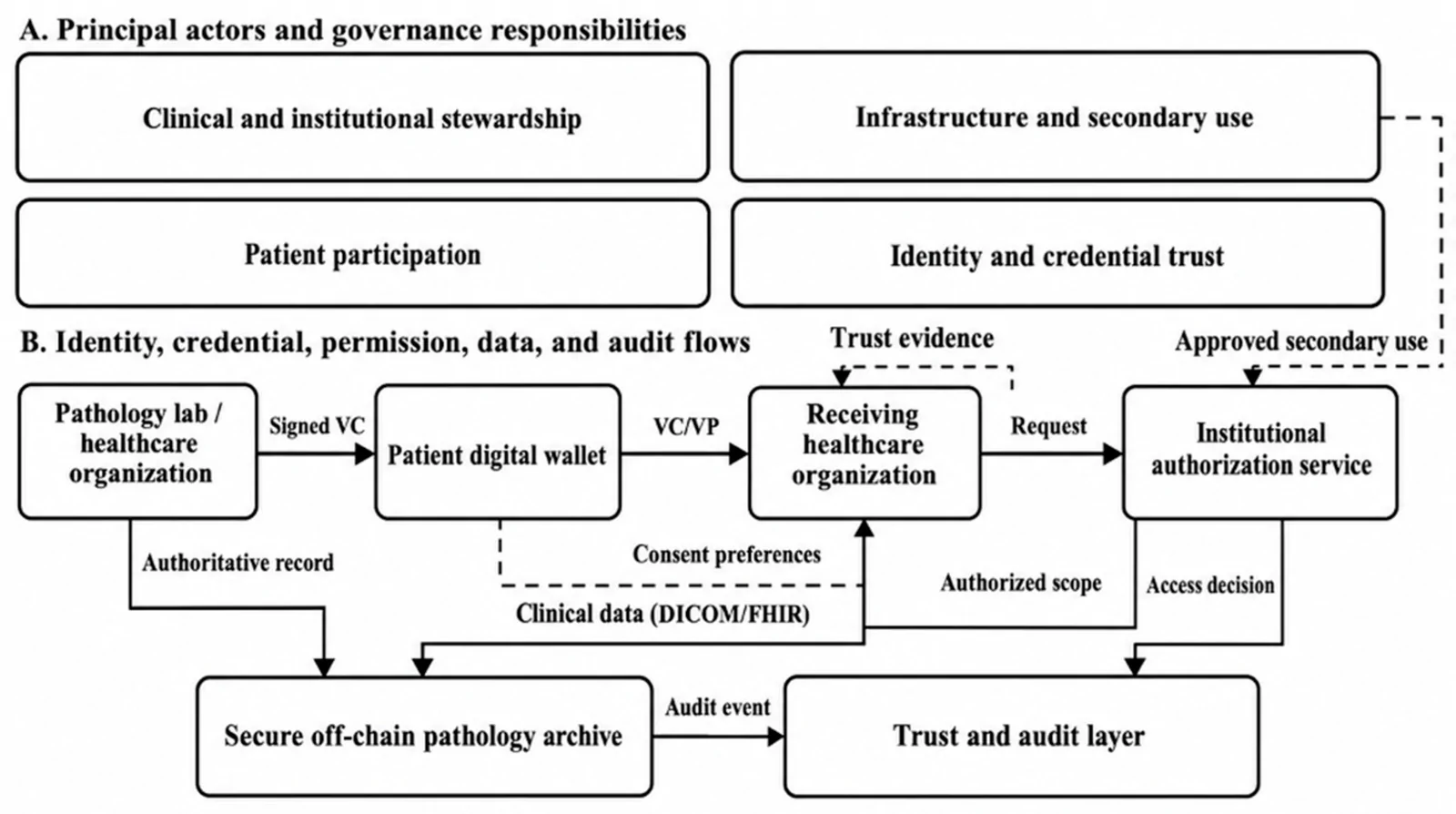
Principal Actors, Governance Responsibilities, Credential Relationships, Authorization Decisions, and Data Flows in the Proposed SSI-Based Digital Pathology Model.

Researchers and secondary users may access only project-authorized data under applicable ethical, legal, data-minimization, security, and audit controls. AI developers and analytic systems are technical tools rather than governance authorities. Dataset provenance, model and version information, outputs, and validation status must remain traceable. AI-generated outputs require clinical review before they affect the authoritative clinical record. Figure 5 brings these actors together and distinguishes issuer–holder–verifier relationships from institutional authorization, record stewardship, secondary-use governance, trust, and audit functions [7,16,40].

The model keeps clinical data objects separate from identity, authorization, and audit objects. Clinical data objects comprise pathology reports, WSI files generated for diagnostic use, clinically validated annotations, clinical and technical metadata, and molecular findings. Governance and trust objects include VCs, VPs, secure clinical data references, record and credential statuses, authorization statuses, and pseudonymous audit-event references.

The model applies a common governance framework across all information types through identity assurance, purpose, and time-limited authorization, credential status checks, secure referral resolution, and auditable access paths. Controls are maintained in an information class-specific manner. Reports and appendices require authorization, version traceability, and professional accountability. WSI files and their metadata must ensure integrity, retention, and interoperable access. Annotations, derived metrics, and AI-generated outputs carry requirements regarding source and validation status. Reinterpretations should remain traceable to the original record rather than altering it. Therefore, credential and authorization policies should define the targeted information class and implement class-specific controls.

The model distinguishes between primary and secondary clinical uses. Primary use directly supports diagnosis, treatment, consultations, follow-ups, and care. Secondary use encompasses research, AI development and training, external validation, population-level analysis, and other reuses that are not necessary for the immediate care of an individual. Quality assurance activities can be classified into two categories depending on their purpose and legal basis [15,16]. Authorization for clinical care does not automatically authorize a secondary use. An approved secondary-use project must clearly state the responsible organization, permitted purpose, data class, duration, and valid approval or other legal basis. Selective disclosure, authentication status checks, and pseudonymous audit references can support data minimization, transparency, termination or withdrawal, and subsequent investigations [13,44,45]. These mechanisms support governance; however, they do not guarantee legal secondary use, scientific validity, or ethical approval.

### 5.3. Layered SSI-Based Digital Pathology Governance Architecture

Figure 5 brings together the principal actors and separates credential exchange, institutional authorization, authorized clinical-data access, and audit flows. VC/VP verification establishes evidence about the issuer, credential status, and integrity but does not itself authorize clinical access. The receiving organization makes the access decision according to role, purpose, scope, duration, consent or another valid legal basis, and institutional policy [15,44,45]. The model comprises four governance layers: SSI, authorization, and consent; secure off-chain clinical data; data sources and interoperability; and blockchain-based trust and audit.

**Layer 1—SSI, Authorization, and Consent Governance:** This layer integrates decentralized identifiers with VCs and VPs. It also encompasses the patient digital wallet, VC status management, purpose- and time-limited authorization, access revocation, emergency access policies, and governance rules. Clinical data themselves are not stored in the patient digital wallet. VCs related to clinical records and secure data references are stored in the wallet. VPs are generated and presented when necessary. Access requests, authorization information, and access history are viewed and tracked through the wallet [13,44,45,46].

**Layer 2—Secure Off-Chain Clinical Data:** Pathology reports, WSI files, annotations, clinical metadata, and molecular findings are stored securely. Access to clinical content is restricted according to verified credentials and the authorization decisions made in Layer 1 [26,41].

**Layer 3—Clinical Data Sources and Interoperability:** This layer encompasses hospital information systems, laboratory information systems, picture archiving and communication systems, and digital pathology infrastructures. DICOM and DICOMweb support the management of WSI files and their accompanying image metadata [31]. HL7 FHIR supports the integration of structured clinical data across different health information systems [33].

**Layer 4—Blockchain-Based Trust and Audit:** This layer supports the recording of references to record integrity, timestamps, VC and authorization status, access and revocation events, and audit trails. Clinical content or directly identifiable patient data are not stored on the blockchain. Instead, cryptographic hashes, timestamps, trust and status references, and minimal pseudonymous references to access, revocation, and emergency access events are recorded [13,26,41].

The architecture also supports authorized use scenarios such as second-opinion consultations, oncological follow-up, treatment planning, molecular assessment, and research applications where the necessary ethical and legal requirements are met. In these scenarios, access is managed in accordance with relevant identity verification, consent, authorization, and validation rules.

### 5.4. Authorization, Consent-Based Access, and Authentication Workflow

**Figure 6:**
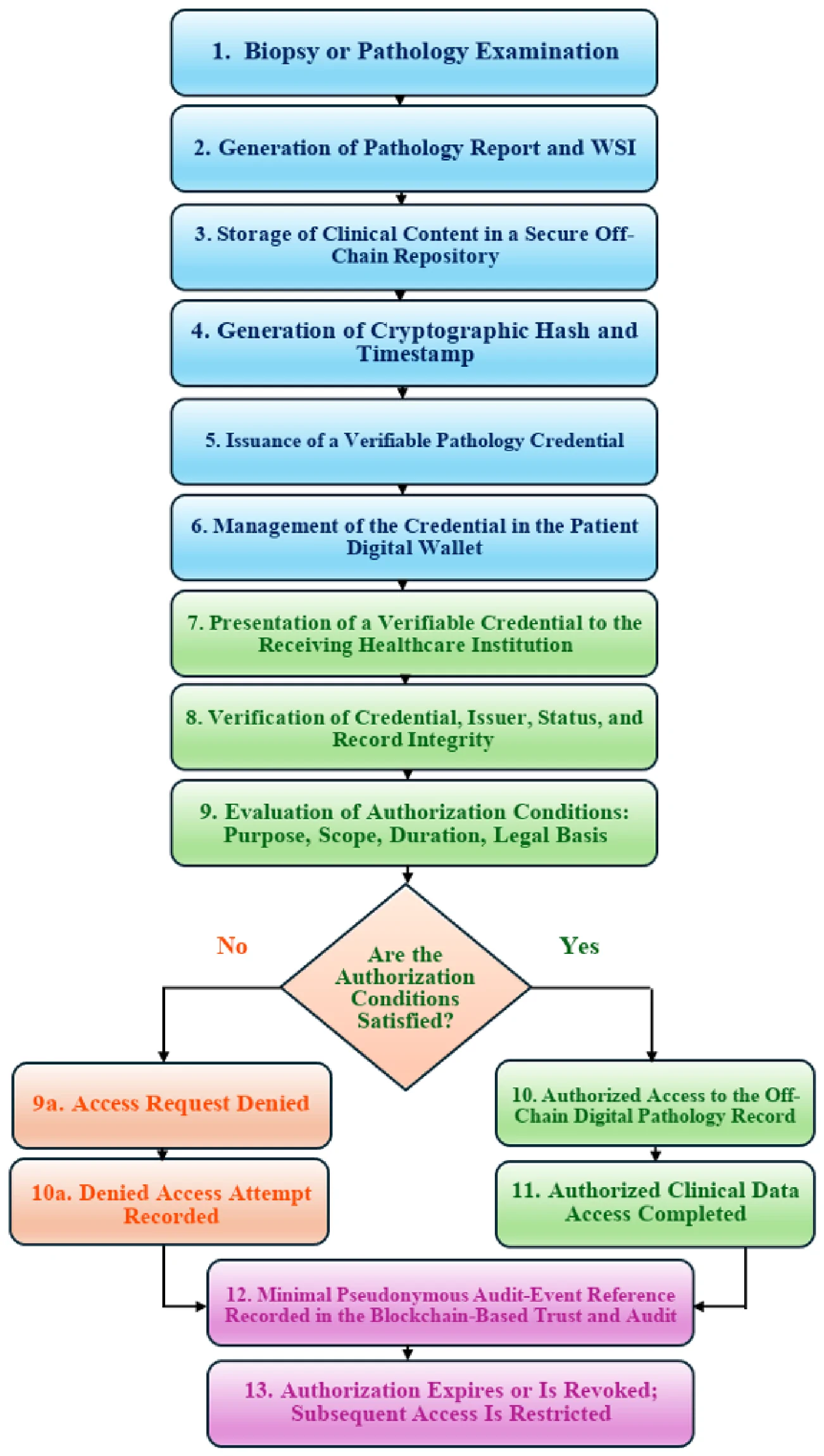
Authorization, Consent, Credential Verification, Access Control, and Audit Workflow for Digital Pathology Records.

Figure 6 illustrates the model workflow for authorization, consent-based access, and authentication processes. The process sequentially begins with clinical record creation and VC issuance, followed by credential verification and a separate authorization assessment. It then records authorized or denied access and the corresponding audit result. Blue represents the creation and management of the credential. Green represents access and verification; orange represents authorization and denied access; and purple represents audit, cancellation, and termination.

First, a biopsy sample is taken, or a pathology examination is completed (Step 1). A pathology report and WSI file are generated (Step 2), and the clinical content is stored in a secure off-chain environment (Step 3) [26,41]. Then, a cryptographic hash value and timestamp are generated (Step 4). An authorized healthcare institution issues a pathology record VC managed in the patient’s digital wallet (Steps 5–6) [44,45].

In the second stage, the patient sends the pathology-record VC, or a VP derived from it to the receiving healthcare institution (Step 7). The institution verifies the digital signature and whether the issuer is trusted and authorized. It then checks the VC’s validity period and revocation status, together with the clinical record’s integrity reference (Step 8) [44,45].

Authentication does not automatically authorize access to clinical data. The access decision is based on the verified identity and professional role of the requester. The assessment also includes the scope of the data, the authorization period, the approval or other valid legal basis, and the institutional access policy (Step 9) [15,16,17].

If the authorization conditions are met, access is granted within the permitted scope (Step 10) and the authorized clinical data access is completed (Step 11). Otherwise, the request is rejected (Step 9a) and the denied access attempt is recorded (Step 10a).

A minimal pseudonymous audit-event record relating to successful or denied access is recorded in the blockchain-based trust and audit layer (Step 12) [13,26,41]. When the authorization expires or is revoked, subsequent access is restricted (Step 13). This stage does not eliminate healthcare institutions’ legal record-retention obligations, the institutional integrity of clinical records, or the existence of data previously accessed lawfully [15,16,17].

Authentication generates evidence of an individual’s or organization’s identity and role through trusted organizational processes and credentials. Neither a DID nor a VC can independently verify a person’s identity or grant access [13,44,45]. Authorization establishes whether a verified actor can perform a specific action within the framework of their role, purpose, scope, duration, legal basis, and organizational policy. Consent is one of the possible inputs that can be included in the authorization decision and does not automatically grant access [15,16,17]. Access control enforces decisions, and auditability records minimal pseudonymous evidence relating to access events. Investigations link organizational records, credential statuses, authorization decisions, and audit events to support dispute reconstruction, abuse investigation, and post-incident reviews [56,57].

### 5.5. Design Scope and Limitations of the Model

The proposed model conceptually describes the fundamental clinical processes and management mechanisms. The model is not based on a specific blockchain platform or a particular software application. Deployment choices should consider the national health infrastructure, existing legislation, organizational requirements, interoperability needs, and security conditions.

If a digital wallet is lost or becomes inaccessible, the patient’s identity must be reverified. Following successful verification, the necessary VCs may be reissued by authorized issuers. Credentials, keys, or authorization references associated with the previous wallet may also be revoked or deactivated through controlled recovery processes. These processes must be supported by additional security controls to prevent identity theft, unauthorized credential issuance, and account-recovery attacks [21,44,45].

The model does not treat consent as the sole or absolute legal basis for data processing. The provision of clinical services, legal obligations, public health, emergencies, and other legal grounds established by applicable legislation must also be considered during authorization [15,16,17]. Accordingly, access decisions cannot be based solely on patient consent. Nor does the model envisage executing all authorization decisions through smart contracts. The approach is intended to allow sensitive decision variables—such as professional role, legal basis, clinical context, and the scope of the requested data—to be evaluated within secure off-chain policy services. The blockchain layer is limited to cryptographic integrity verification, timestamping, status references, and minimal audit trails [13,26,41].

Long-term clinical memory requires sustainable governance throughout ongoing technological and institutional changes. Throughout the lifecycle, institutions maintaining pathology records may merge or cease operations. Furthermore, technology providers, identity authorities, identifiers, keys, credential formats, and interoperability standards may change. Therefore, the model envisages persistent storage, version histories, explicit export formats, designated successor custodians, key and credential transfer, trust-registry updates, and regular transition tests. The aim of these arrangements is to ensure that the origin, integrity, access decisions, and audit trail of the record remain interpretable even if the original technical environment disappears [5,44,45]. However, the effectiveness of these mechanisms has not been established by the current conceptual assessment and requires long-term technical, institutional, and regulatory validation.

### 5.6. Implementation Challenges and Equity Safeguards

The conceptual scope alone does not guarantee viable and equitable implementation. Therefore, the implementation should be phased in. It should not be assumed that patients have a compatible smartphone, reliable connection, or ability to manage cryptographic credentials and complex consent settings [20,35,58]. Limited digital skills, language barriers, visual or cognitive limitations, and low trust in digital systems can make wallet use difficult. Access only through smartphones can also exacerbate existing inequalities [35,58]. Therefore, simple and multilingual interfaces, accessibility tests, assisted registration, and trained help desks can be offered. Alternative access channels, such as web, kiosks, call centers, and face-to-face services, can be provided. Legally bounded representative access is needed for patients who cannot manage their wallets independently [22,37]. Recovery processes should include identity re-verification, credential reissuance, revocation of compromised credentials or keys, and fraud monitoring. Secure non-digital alternatives should be offered for device loss or connectivity failure [20,22].

Healthcare organizations need technical and organizational capacity within the scope of identity verification, identity document lifecycle management, DICOM/DICOMweb and FHIR integration, audit monitoring, and shared trust policies [12,31,32]. Before scaling up, organizations should conduct phased pilot applications and perform compliance tests; prepare procurement guidelines with shared issuer records and secure targeted funding. Pathology, information management, cybersecurity, records management, and patient support teams should have defined responsibilities and training requirements. Authorization, remediation, and recovery activities should be integrated into clinical workflows [11,12]. The main risks are wallet security breaches, erroneous authorization, interruptions in verification services, misuse of emergency access, and ambiguity of liability [25,40]. Managing these risks requires data protection impact assessments, least privilege policies, incident response procedures, periodic audits, assignment of responsibilities, and post-incident investigation [16,56,57]. Public authorities should define the legal and trust framework; organizations should maintain authorized records and controls; technology providers should verify compliance with standards; and patient representatives should contribute to accessibility and equity audits. During the scaling process, predefined safety, usability, accessibility, equity, and continuity of care outcomes should be monitored [11,20,58].

## 6. Internal Requirement-Coverage Assessment

Traditional, centralized digital, and SSI-based workflows were compared within the R1–R11 framework using the internal requirement-coverage assessment method defined in Section 3.4.2. Table 7 summarizes whether each workflow specifies mechanisms corresponding to each requirement.

In the proposed SSI-based scenario, the mechanisms corresponding to R1–R11 are defined at the conceptual level. Implementation depends on compliance with standards, institutional integration, trust management, identity and access management, workforce capacity, funding, and adherence to applicable laws. The evaluation addresses the fulfillment of internal requirements and maintenance of conceptual consistency. It does not provide any evidence regarding the technical safety, clinical applicability, patient safety implications, usability, or real-world performance of the model.

Table 7 indicates that traditional and centralized digital pathology scenarios may provide various mechanisms related to patient access, record visibility, and institutional data management. However, cross-institutional verifiable sharing, granular authorization, selective disclosure, and patient-visible access management may remain limited depending on the technical infrastructure used and institutional governance rules.

### 6.1. External Benchmark-Oriented Assessment

To mitigate circular reasoning in the R1–R11-based assessment, selected domains from the NIST Privacy Framework and NIST Cybersecurity Framework 2.0 [56,57] were applied in a supplementary post hoc assessment. These external criteria were not used to derive the requirements or design the model. The assessment was conceptual and did not constitute formal compliance testing, certification, or independent validation. Traditional workflows largely leave governance to individual institutions, whereas centralized digital systems retain platform-level control. The proposed model shows stronger conceptual alignment by explicitly defining issuers and verifiers, trust policies, authorization responsibilities, and auditable access. Wallets, authorization status, access histories, and revocation mechanisms also support participation and transparency. Comprehension, accessibility, assisted authorization, and representative access still require empirical usability and equity assessment.

The model demonstrates conceptual alignment with external frameworks in terms of data protection, least privilege, detection, response, and recovery [56,57]. Secure off-chain storage, minimal data sharing, and restricted authorization support data-protection principles. However, the effectiveness of key management and security controls has not been tested.

The model supports the auditing of successful, denied, revoked, and emergency access events. However, monitoring thresholds, incident-response performance, staffing requirements, and organizational accountability outcomes remain unvalidated. Furthermore, while credential reissuance and wallet recovery are defined, service restoration, device loss, cross-institutional continuity, and system failure scenarios have not yet been tested.

### 6.2. Model Scope and Future Research

The proposed model specifies governance functions by separating issuers, holders, verifiers, and institutional stewards. It includes verifiable presentations, credential-status checks, policy-based authorization distinct from authentication, secure off-chain access, and minimal audit references [13,44,45]. These mechanisms describe a conceptual architecture rather than a deployed system or demonstrated outcome. Under appropriate legal and institutional conditions, they could support patient-visible authorization, verifiable cross-institutional sharing, lifecycle accountability, and transparent secondary use [15,31,32]. These are conditional design propositions rather than empirical findings.

Building on the implementation challenges outlined in Section 5.6, future research should test interoperability, legacy-archive migration, wallet recovery, institutional readiness, and legal accountability. Phased pilots should evaluate safety, usability, equity, cost, workflow effects, continuity, and failure recovery [15,20,32,58].

## 7. Conclusions and Recommendations

This study correlates patient-reported access difficulties and complementary governance evidence with a traceable set of requirements for digital pathology archives. Requirements R1–R11 form the basis of the SSI model developed using DSR. These requirements are derived from sources based on complaints from Şikayetvar, NHS Written Complaints data, the academic literature, policy and regulatory documents, and technical standards. The model separates clinical content from identity, verification, authorization, and auditing functions. While clinical content remains in secure off-chain environments, blockchain layer integrity verification is limited to timestamps and minimal references to the authorization status with credentials. A valid VC alone does not authorize access to the clinical data. Access is assessed separately based on role, purpose, scope, duration, approval, and other applicable legal grounds. The assessment of the internal requirement-coverage assessment revealed that traditional and centralized digital workflows may include relevant mechanisms. However, verifiable cross-institutional sharing, granular authorization, selective disclosure, and patient-traceable access management depend on infrastructure and institutional policies. The supplementary NIST-based post hoc assessment provides an external conceptual cross-check. Neither assessment independently validates technical security, clinical applicability, usability, or institutional performance.

Resource-requirement mapping has shown that not all requirements stem directly from complaint frequencies. Specifically, R11 was primarily derived from regulatory and ethical sources, as well as internal clinical reviews.

Future work should translate conceptual consistency into phased empirical validation through security, confidentiality, interoperability, usability, and accessibility testing. Multicenter pilot studies should assess the clinical workflow, cost, institutional readiness, continuity of care, authorization failures, urgent access, and equity outcomes.

This study’s contribution is twofold. First, it conceptualizes digital pathology archives as long-term clinical memory that preserves context, provenance, integrity, version history, and governed reuse. Second, it proposes an SSI-based governance approach for managing identity assurance, authorization, access, provenance, credential and record status, and accountability over time while institutions retain authoritative stewardship of clinical records. Together, these contributions provide a testable governance foundation rather than a production-ready or empirically validated system.

### Policy and Implementation Pathway

Four complementary policy options are proposed to translate R1–R11 into implementable policy actions. Each option defines the responsible actors, expected benefits, costs, feasibility conditions, and equity safeguards [59,60,61].

**1. Inclusive trust and accountability:** Health authorities and regulators should identify authorized credential issuers and verifiers. Liability allocation, credential status, emergency access, retention of audit records, and institutional participation requirements should also be clearly defined. This common framework can reduce bilateral trust negotiations and institutional differences by providing consistent governance for R1 and R5–R11. Coordination and legal-design costs are high, but phased participation and national guidance provide moderate feasibility. Equity safeguards should include representatives of patients, people with disabilities, rural care, pathology, privacy, and cybersecurity. Smartphone-only access should be avoided.

**2. Standards-based pilots:** Health authorities and funders should support selected pathology networks in testing DICOM/DICOMweb, FHIR, DID/VC, wallet recovery, and cross-institutional authorization. These pilots should generate implementation evidence for R1–R10 and identify workflow, interoperability, conformance, security, and cost barriers before scale-up. Initial costs may be moderate to high, so sites should include urban, rural, and differently resourced institutions. Assisted access should be funded, and differential failure rates across patient groups should be assessed.

**3. Inclusive access and authorization:** Healthcare institutions should offer accessible web and mobile interfaces, in-person or call center support, and non-digital alternatives to access health information. This can improve usability and equity for R1, R2, R7, and R9 without delegating institutional record-keeping responsibilities to the patient. The main disadvantages are recurring staffing and training costs. Services are feasible when they are integrated into existing patient support and record management processes. Safeguards should include accessibility and multilingual testing, protection of non-wallet users, and regular equity audits of the program.

**4. Assurance and staged assessment:** Regulatory bodies and healthcare organizations should require the reporting of privacy and security impact assessments, compliance testing, incident response plans, as well as emergency access reviews and pilot results in a predetermined manner. This approach supports R3, R6, and R8–R11, thereby reducing the risk of misinterpreting conceptual alignment as a validated indicator of safety or effectiveness. The costs associated with the assurance provided are moderate, and the feasibility of implementation is high when requirements are aligned with procurement and accreditation cycles. Aggregate safety and equity results should be reported, and unsafe components should be suspended.

A pragmatic process involves establishing governance and assurance principles, conducting pilot studies on limited interoperability and usability, and scaling up after the previously defined criteria for safety, clinical workflow, continuity, accessibility, cost, and equity are met.

## Figures and Tables

**Table 1:** Functions of Data and Document Sources in the Study.

Source	Source Type	Form of Analysis/Review	Function in the Study
Şikayetvar “E-Nabız Pathology Result” complaints	Publicly available complaint texts	Codebook-based thematic content analysis	Identification of patient-reported problem areas
NHS Written Complaints data	Aggregated official complaint statistics	Category and trend analysis	Illustrative external governance context; no cross-country prevalence comparison or corroboration
Academic literature	Conceptual and technical studies	Conceptual and technical synthesis	Establishment of the theoretical foundation and design implications
Policy and regulatory documents	Documents related to the GDPR, KVKK, and EHDS	Governance-focused document review	Identification of legal principles and design boundaries
Technical standards and specifications	DICOM/DICOMweb, HL7 FHIR, W3C DID, and VC	Standards–requirements mapping	Identification of interoperability, verifiability, and model components

**Table 2:** Inclusion and Exclusion Criteria for the Şikayetvar Corpus.

Inclusion Criteria	Exclusion Criteria
Related to a pathology result, record, or associated digital access process	Not directly related to pathology
Contains at least one issue involving access, visibility, delay, sharing, record integrity, or data control	Related solely to appointments, fees, physical services, or staff conduct
Publicly available, accessible, and sufficiently detailed for thematic content analysis	Duplicate, withdrawn, inaccessible, or insufficiently detailed for coding
Can be safely de-identified	Cannot be safely de-identified for analysis or reporting

**Table 3:** T1–T11 Thematic Content Analysis Codebook for Şikayetvar Complaints.

Code	Theme	Operational Definition	Governance Implication
T1	Pathology result not visible or accessible	The pathology result or report is not visible in the e-Nabız system, or the relevant record cannot be accessed.	Strengthening patients’ timely access to pathology records
T2	Record not visible despite notification	A notification indicates that the result is ready; however, the relevant result or report is not visible in the system.	Ensuring consistency between notifications and record status, as well as record visibility
T3	Delayed availability of the result in the system	Although the pathology examination has been completed by the healthcare institution, the result or report is not made available in the system in a timely manner.	Supporting timely access and continuity of care
T4	Difficulty accessing related tests and diagnostic records	Laboratory tests, imaging examinations, or other diagnostic records associated with the pathology record cannot be accessed.	Ensuring integrated access to related tests and diagnostic records
T5	Record missing, incomplete, or inaccessible	The pathology record cannot be found in the system, is displayed incompletely, or the relevant file cannot be opened.	Ensuring record integrity and the traceability of the record lifecycle
T6	Disruption of continuity of care, second-opinion, or treatment processes	The inability to access the pathology result adversely affects follow-up, referral, second-opinion, or treatment processes.	Supporting record portability and continuity of care
T7	Cross-institutional record visibility or sharing problem	The pathology record cannot be viewed or used by another physician or healthcare institution.	Improving cross-institutional record sharing and interoperability
T8	Access restriction on privacy grounds	Access to or sharing of the pathology record is restricted on privacy or confidentiality grounds.	Establishing an appropriate balance between privacy protection and access requirements
T9	Technical, usability, or communication problem	Problems arise when using the web or mobile application, locating records, or communicating with the relevant healthcare institution.	Supporting reliable and usable digital access
T10	Patient request for access to and control over personal records	The patient requests access to or the ability to view their pathology records or participate in decisions concerning their sharing.	Strengthening patient participation and meaningful control over decisions concerning record access and sharing
T11	Request for correction or resolution	The complainant explicitly requests a correction, explanation, or prompt resolution regarding a record or access problem.	Making correction and resolution processes visible and traceable

**Table 4:** Evaluation of Selected NHS Complaint Categories from a Digital Pathology Governance Perspective.

NHS Complaint Category	2022–2023	2023–2024	2024–2025	Governance Significance for Digital Pathology	Related Requirement
Communications	47,301	51,403	56,180	Need for timely and reliable information on record status and the results process	R2, R9
Confidentiality/ Privacy and Dignity	5446	5596	5831	Limiting access to clinical data based on privacy, purpose, and scope	R6
Delay in Diagnosis/Failure to Refer	4953	5580	6617	Timely transfer of results to diagnostic, referral, second opinion, and treatment processes	R2, R5
Records: Inaccurate/Incorrect	1719	2232	3112	Traceability for record integrity, correction, and versioning	R3, R8
Refusal to Allow Access to Records	186	226	279	Supporting patients’ access to their own health records	R1
Records: Loss of Records	182	225	216	Ensuring traceability of record integrity and lifecycle	R3
Clinical Treatment: Pathology Group	206	267	336	Managing pathology records in an integrated and image-linked manner	R4

**Table 5:** Source-to-Requirement Traceability for R1–R11.

Code	Şikayetvar	NHS Context	Literature/Regulatory	Technical/Clinical Contribution	Governance Requirement
R1	T1, T10	Refusal of access to records	EHDS, GDPR, KVKK access boundaries	Wallet and VC-mediated access design	Verifiably accessible long-term clinical memory components
R2	T2, T3	Communications; diagnostic/referral delay	Timely access and continuity principles	Timestamped record and credential-status linkage	Verifiable record status with timestamped tracking
R3	T5	Inaccurate and lost records	Integrity, provenance, and accountability literature	Hashes, version relations, and audit evidence	Record integrity and an auditable record lifecycle
R4	T4	Pathology-service category	Digital pathology archive literature	DICOM/DICOMweb and HL7 FHIR	Integrated, image-linked, interoperable archive
R5	T6, T7	Diagnostic/referral delay	Continuity-of-care literature	Interoperable references and verifiable sharing	Portable, verifiable cross-institutional sharing
R6	T8	Privacy and dignity	GDPR, KVKK, EHDS	Off-chain policy service and granular authorization	Access limited by purpose, scope, duration, and minimum necessity
R7	T10	No directly equivalent selected category	e-Nabız permissions and dynamic-consent literature	Wallet-based consent and authorization-status management	Dynamic, time-limited, and revocable consent
R8	T5, T11	Inaccurate records	Clinical correction and accountability principles	Record versioning and VC status management	Correction and version tracking linked to credential status
R9	T9, T11	Communications	Administrative accountability literature	Request-status tracking and auditable events	Auditable request and resolution process
R10	T8, T10	Privacy and dignity	Minimum-necessary and privacy principles	W3C VC/VP and selective-disclosure mechanisms	Verification using the minimum necessary data
R11	No direct complaint-frequency basis	No directly equivalent selected category	Regulatory and ethical duties concerning emergency access	Internal clinical review of an auditable break-glass design	Auditable emergency access and post-incident review

**Table 6:** Requirement–Model Traceability Matrix.

Code	Governance Requirement	Model Layer/Component	Proposed Governance Mechanism
R1	Verifiable access to components of long-term clinical memory with patient-managed access	Patient digital wallet, DID, VC/VP, and secure data access components	Issuance of a VC for the pathology record by an authorized healthcare institution; management of this credential by the patient in a digital wallet and access to the relevant record under authorization conditions
R2	Verifiable record status and timestamped tracking	Clinical record lifecycle, VC status management, timestamps, and audit layer	Separate but linkable tracking of clinical record status, VC validity, timestamps, and related lifecycle events
R3	Record integrity and an auditable record lifecycle	Secure off-chain clinical data layer, cryptographic hashes, provenance and version relationships, and audit layer	Verification of record integrity through cryptographic hashes; tracking of record provenance, corrections, supplementary reports, and version changes
R4	Integrated, image-linked, and interoperable digital pathology archive	Clinical data sources and interoperability layer	Linking pathology reports, WSI files, annotations, and associated clinical and technical metadata through secure DICOM/DICOMweb- and HL7 FHIR-based data references
R5	Portable and verifiable cross-institutional record sharing	DID verification, VC/VP sharing, secure data access, trust framework, and interoperability components	Verification of the issuer’s authority, record source, and VC status; secure and interoperable sharing of authorized clinical content across institutions
R6	Access based on data minimization and limited by purpose, scope, and duration	SSI, authorization and consent governance layer, and off-chain policy service	Evaluation of access decisions based on the requesting actor’s identity and role, access purpose, legal basis, data scope, access duration, and the minimum necessary data principle
R7	Dynamic, time-limited, and revocable consent	Patient digital wallet, consent and authorization status, representative access, and consent withdrawal components	Granting consent, updating its scope and duration, managing it through an authorized representative, and withdrawing it with respect to future consent-based access
R8	Clinical record correction and version tracking with credential status management	Clinical record source, authorized healthcare institution, VC status management, version relationships, and audit layer	Creation of corrections and supplementary reports by the authorized healthcare institution, preservation of previous versions, and revocation and reissuance of the relevant VC when necessary
R9	Auditable request and resolution process	Patient digital wallet, request and status management functions, authorization policy service, and audit trail	Receipt of access, error, and correction requests; traceable recording of request statuses, responses provided, and actions performed
R10	Verification using the minimum necessary data through selective disclosure	VC/VP component, selective disclosure mechanism, and verification policy	Disclosure of only the claims or data fields required for a specific verification purpose, provided that the VC format used supports this functionality
R11	Auditable emergency access and post-incident review	Emergency access policy, professional role verification, authorization service, and audit layer	Provision of justified exceptional access based on professional role verification, the minimum necessary data, time limitation, and a mandatory audit record, followed by post-incident review

**Table 7:** Internal Conceptual Coverage of R1–R11 across Reference Pathology Workflows.

Code	Governance Requirement	Scenario 1: Traditional Pathology Workflow	Scenario 2: Centralized Digital Pathology Workflow	Scenario 3: Proposed SSI-Based Workflow	Primary Implementation Condition
R1	Verifiable access to components of long-term clinical memory with patient-managed access	Partially addressed	Partially addressed	Conceptually addressed	Ensuring the availability of usable digital wallets, secure identity recovery processes, and institutional participation
R2	Verifiable record status and timestamped tracking	Partially addressed	Partially addressed	Conceptually addressed	Consistently linking the clinical record lifecycle with VC statuses
R3	Record integrity and an auditable record lifecycle	Partially addressed	Partially addressed	Conceptually addressed	Storing clinical content in secure off-chain environments and verifying its integrity through cryptographic hashes
R4	Integrated, image-linked, and interoperable digital pathology archive	Not defined	Partially addressed	Conceptually addressed	Ensuring interoperability compliant with DICOM/DICOMweb and HL7 FHIR standards
R5	Portable and verifiable cross-institutional record sharing	Partially addressed	Partially addressed	Conceptually addressed	Establishing a common trust framework and a registry of authorized VC issuers
R6	Access based on data minimization and limited by purpose, scope, and duration	Partially addressed	Partially addressed	Conceptually addressed	Establishing implementable and interoperable authorization policies across institutions
R7	Dynamic, time-limited, and revocable consent	Not defined	Partially addressed	Conceptually addressed	Clearly distinguishing consent from other legal bases and managing the consent lifecycle
R8	Clinical record correction and version tracking with credential status management	Partially addressed	Partially addressed	Conceptually addressed	Linking record corrections and addendum reports with VC revocation and reissuance processes
R9	Auditable request and resolution process	Partially addressed	Partially addressed	Conceptually addressed	Integrating status management for access, error, and correction requests into institutional processes
R10	Verification using the minimum necessary data through selective disclosure	Not defined	Partially addressed	Conceptually addressed	Adopting VC/VP formats and verification mechanisms that support selective disclosure
R11	Auditable emergency access and post-incident review	Partially addressed	Partially addressed	Conceptually addressed	Defining emergency access roles, access conditions, limits on duration and data scope, and post-incident review processes

## Data Availability

Source complaints are publicly accessible as long as they remain available on the Şikayetvar platform. The aggregated findings are presented in the manuscript. The complaint-level coding matrix has not been made public to prevent the re-association of coded records with original complaints. Additional aggregated data that do not contain complaint-level information may be obtained from the corresponding author upon reasonable request.

## Abbreviations

AI, artificial intelligence; DID, decentralized identifier; DICOM, Digital Imaging and Communications in Medicine; DSR, Design Science Research; EHDS, European Health Data Space; FHIR, Fast Healthcare Interoperability Resources; GDPR, General Data Protection Regulation; HIS, hospital information system; KVKK, Personal Data Protection Law No. 6698; LIS, laboratory information system; NHS, National Health Service; PACS, picture archiving and communication system; SSI, self-sovereign identity; VC, verifiable credential (plural: VCs); VP, verifiable presentation (plural: VPs); W3C, World Wide Web Consortium; WSI, whole-slide imaging; WSI file, whole-slide image file.

## Appendix A

Structured Internal Clinical Expert Review and Summary of Model Refinement.

**Table A1:** Structured Internal Clinical Expert Review and Summary of Model Refinement.

Evaluation Dimension	Focus of Review	Affected Model Element	Clarification Incorporated into the Model
Clinical terminology	Conceptual distinction among pathology records, clinical content, health data, and governance data	Conceptual definitions and data layers of the model	Pathology reports, WSI files, clinical and technical metadata, and governance and trust objects were distinguished from one another.
Actors and responsibilities	Clarity of the roles of the pathologist, pathology laboratory, healthcare institution, patient, recipient healthcare professional, and research institution	Actors and roles	Responsibility for creating and correcting clinical records was assigned to authorized healthcare institutions and clinical actors. It was clarified that patient control does not entail authority to modify the content of clinical records.
Pathology report–WSI relationship	Joint interpretability of pathology reports and the associated WSI files	Clinical data sources and interoperability layer	The model provides for linking pathology reports and the associated WSI files through secure data references based on DICOM/DICOMweb and HL7 FHIR.
Clinical data objects	Identification of the data objects considered clinical content within the model	Secure off-chain clinical data layer	Pathology reports, WSI files generated for diagnostic use, clinically validated annotations, clinical and technical metadata, and molecular findings were defined as clinical data objects.
Separation of clinical content and governance data	Separation of clinical records from VCs, VPs, authorization statuses, and audit references	Layered governance architecture	Clinical content was retained in secure off-chain environments, while blockchain use was limited to cryptographic hashes, timestamps, status references, and minimal audit records.
Record lifecycle	Distinction between clinical record status and VC status	Record and credential status management	The clinical record lifecycle and the validity, suspension, or revocation status of the VC were defined as separate but linkable processes.
Correction and reissuance	Management of corrections and addendum reports relating to clinical records	Versioning and audit mechanisms	Instead of allowing patients to modify their clinical records, this model ensures the traceability of previous versions by requiring corrections and additions to be prepared as new versions by an authorized healthcare institution.
VC revocation and reissuance	Management of the status of a VC associated with a corrected clinical record	VC status management	When a clinical record is corrected or reissued, the associated VC may be revoked where necessary, and a new VC may be issued for the current version of the record.
Second opinion and continuity of care	Reuse of pathology records by different institutions and clinicians	Authorization and sharing workflow	Second opinions, referrals, oncological follow-up, molecular evaluation, and treatment planning were incorporated into the model as distinct authorized contexts of use.
Authorized representative access	Limits of the representative’s role when the patient is unable to manage access	Consent and representative access components	Representative access was limited by the scope of authorization, purpose of access, and duration. The model provides for such access to be revocable and auditable.
Emergency access	Access in exceptional clinical circumstances in which patient consent cannot be obtained	Emergency access policy and audit layer	Emergency access was made conditional on professional role verification, justification, the minimum necessary data, time limitations, mandatory audit logging, and post-incident review.
Risks related to clinical feasibility	Alignment of the model with existing clinical workflows, patient safety, and institutional responsibilities	Design scope and limitations of the model	It was clarified that credential verification does not automatically provide access to clinical data. Access decisions must be evaluated separately according to clinical context, professional role, purpose, scope, duration, consent, and other legal bases.

Note. This table summarizes the structured internal clinical expert review conducted as part of the DSR-based model refinement process. The technical standards and model components referenced in the table are described and cited in Section 2 and Section 5. The review constitutes an internal model-refinement activity rather than an independent external validation.

## Appendix B

Detailed Rationale for the Internal Requirement-Coverage and Conceptual Consistency Assessment.

**Table A2:** Rationale for the Internal Requirement-Coverage Assessment of R1–R11 Requirements.

Code	Traditional Workflow	Centralized Digital Workflow	Proposed SSI-Based Workflow
R1	Access through institutional requests; institution-dependent sharing	Portal access; platform-dependent scope and control	Verifiable access through a digital wallet and VC/VP
R2	Institutional or manual tracking; limited patient visibility	Status and time display; limited cross-institutional linkage	Linkable record and VC statuses, timestamps, and lifecycle events
R3	Institutional integrity; limited versioning and cross-institutional verification	Backup and versioning; infrastructure-dependent verification	Secure off-chain storage and cryptographic integrity verification
R4	No image-linked, interoperable archive	Digital archive; infrastructure- and vendor-dependent linkage	Report–WSI linkage through DICOM/DICOMweb and HL7 FHIR
R5	Physical or request-based sharing with limited verifiability	Electronic but platform-limited sharing	DID, VC/VP, a shared trust framework, and secure access
R6	Institutional and manual permissions; limited granular policies	Role-based access but limited cross-institutional policies	Access based on purpose, scope, duration, role, legal basis, and the minimum necessary data
R7	Static consent; no dynamic lifecycle	Sharing permissions; limited scope and withdrawal	Dynamic, time-limited, representative-mediated, and revocable consent
R8	Institutional correction; limited version tracking	Correction and versioning; limited linkage to VC status	Linkage among correction, versioning, VC revocation, and reissuance
R9	Institutional requests; limited end-to-end tracking	Centralized support; limited linkage to the lifecycle and audit trail	Traceable request, response, and audit process
R10	No selective disclosure mechanism	Field- or role-based restrictions; limited VC/VP support	Selective disclosure of necessary claims or data fields
R11	Institutional emergency access; limited logging and review	Policy-dependent “break-glass” mechanism	Role, justification, minimum necessary data, time limits, audit, and post-incident review

This table provides a conceptual synthesis of the literature and technical context discussed in Section 2 and Section 3.4.2 and supports the requirement–model traceability analyses presented in Section 5 and Section 6.
